# Tick-Borne Diseases of Humans

**DOI:** 10.3201/eid1111.051160

**Published:** 2005-11

**Authors:** Robert P. Smith

**Affiliations:** * Maine Medical Center, Portland, Maine, USA

**Keywords:** ticks, book review, rickettsia, *Borrelia*, *Babesia*

During the past 2 decades, the scientific landscape of tickborne diseases has changed remarkably. In part because of advances in molecular biology, more than 10 new rickettsial diseases, several ehrlichial diseases, and novel agents of *Borrelia* and *Babesia* genera have been recognized. This renaissance of interest in tickborne infections benefits from advances in molecular phylogenetics and diagnostics, immunology, and informatics that provide tantalizing insights into the complexities of vectorborne infection. Tick-Borne Diseases of Humans is a well-referenced textbook that encompasses these new insights in vector biology and reviews the emerging epidemiology and clinical science of these diseases as they occur across the globe. The editors' goal of providing a "comprehensive" resource is admirably fulfilled.

The book, consisting of 20 chapters by 40 contributors, is divided into 3 sections. The first section includes excellent reviews of tick biology and systematics, tick-pathogen interactions, host responses, and vector management. This section provides a superb overview. While thorough and up-to-date, occasional redundancy occurs between chapters by different authors that could have been streamlined with additional editing. A concise and well-written chapter on the clinical approach to diagnosis and management of these diseases also seems misplaced; it would fit in better at the start of the next section.

Section 2 of the book includes summaries of major and lesser known tickborne infections. These chapters each provide detailed information on specific vectors and pathogens and on the epidemiology and clinical characteristics of the diseases they cause. While the description of the molecular biology and vector ecology of these infections is generally excellent, the clinical discussions often lack the nuance and detail of current infectious diseases texts. Nevertheless, each chapter provides current and well-referenced information on disease manifestations, diagnosis, and treatment. Several chapters, i.e., those on anaplasmosis, relapsing fever, and Lyme borreliosis, are superb in all aspects.

Section 3 includes a series of global maps that depict the distribution of different tick vectors or the diseases they cause. While useful overall, maps on this scale do not convey the focality of tick distribution, and their organization in the text (i.e., mixing of vector maps with disease maps) could be improved. The maps are followed by a color atlas of tickborne diseases with plates depicting typical skin lesions and other clinical findings along with examples of microscopic pathology. This part of the book is visually compelling, although the reproductions of microscopic pathology are often small and therefore difficult to view in detail. Section 3 concludes with an almanac of the geographic distribution of ticks and the diseases they cause. This information is often difficult to find, and its inclusion in a chapter of the text is useful.

In summary, Tick-Borne Diseases of Humans is an excellent resource for a diverse audience. Vector biologists (whether molecular or ecologic in focus), infectious disease physicians, and those involved in the public health surveillance and control of these diseases will find this book to a valuable addition to current texts.

